# Drugs associated with cataract formation represent an unmet need in cataract research

**DOI:** 10.3389/fmed.2022.947659

**Published:** 2022-08-15

**Authors:** Jack Carlson, Kate McBride, Michael O’Connor

**Affiliations:** ^1^School of Medicine, Western Sydney University, Campbelltown, NSW, Australia; ^2^Translational Health Research Institute, Western Sydney University, Campbelltown, NSW, Australia

**Keywords:** prescription drug, dexamethasone, human pluripotent stem cell, micro-lens, cataracts, bioinformatics, lens

## Abstract

Decreased light transmittance through the ocular lens, termed cataract, is a leading cause of low vision and blindness worldwide. Cataract causes significantly decreased quality of life, particularly in the elderly. Environmental risk factors, including aging, UV exposure, diabetes, smoking and some prescription drugs, are all contributors to cataract formation. In particular, drug-induced cataract represents a poorly-addressed source of cataract. To better understand the potential impact of prescription drugs on cataract, we analyzed publicly-available drug prescriptions data from the Australian Pharmaceutical Benefits Scheme. The data was analyzed for the 5-year period from July 2014 to June 2019. Analyses included the number of prescriptions for each drug, as well as the associated government and total prescription costs. The drugs chosen for analysis belonged to any of four broad categories—those with known, probable, possible or uncertain association with cataract in patients. The analyses revealed high prescription rates and costs for drugs in the Known category (e.g., steroids) and Possible category (e.g., psychotropic drugs). Collectively, these data provide valuable insights into specific prescription drugs that likely contribute to the increasing annual burden of new cataract cases. These data highlight the need—as well as new, stem cell-based opportunities—to elucidate molecular mechanisms of drug-induced cataract formation.

## Introduction

Cataracts disrupt light transmission through the lens of the eye. Excluding uncorrected refractive errors, cataracts are the leading cause of blindness and low vision worldwide—with over 65 million (M) patients affected in 2015 (1). Current cataract treatment involves removal of the cataractous lens tissue and replacement of lens function with a synthetic intra-ocular lens. Where access to treatment is available, cataract surgery is a relatively simple and effective approach to restoring vision. However, despite continued advances in cataract surgery, the number of patients affected by cataract continues to increase (1).

Over 70 drugs have been associated with a known or suspected increased risk of cataract formation (2). These drugs can be grouped into four distinct categories based on the evidence underpinning their association with cataract formation: (i) Known category drugs are known to increase the risk of cataract in patients; (ii) Probable category drugs are likely to cause increased risk of cataracts; (iii) Possible category drugs, and (iv) Uncertain category drugs may increase cataract risk but the data is inconclusive (Supplementary Table 1). A collective analysis of the prescribing rates for the cataract-associated drugs in these four categories is yet to be performed.

In Australia, the Federal Government manages two programs that provide universal healthcare to citizens, permanent residents, and some international travelers. The Medicare scheme provides access to health and hospital services, including cataract surgery, at low or no cost. The Pharmaceutical Benefits Scheme (PBS) subsidizes the cost of prescription medicines approved for inclusion in the scheme. Among the PBS-listed medicines are drugs known or suspected to increase the risk of cataract. To investigate the number of prescriptions and associated costs for these drugs in Australia, we examined publicly available, PBS drug prescriptions data for the 5-year period from July 2014 to June 2019.

## Results

### Known category drugs dominated by high and increasing glucocorticoid prescriptions

Of the 31 drugs known to increase the risk of cataract (Supplementary Table 1), analysis of the PBS data revealed 17 were prescribed over the 5-year period analyzed (Table 1). The top 3 most prescribed Known category drugs averaged more than 1 M prescriptions per year, and cost from $18 M to $53 M a year each (Table 1).

**TABLE 1:** PBS data (2014–2019) showing prescription numbers and costs for members of the known category of drugs associated with cataract (gray background = steroids).

Drug name	Total prescriptions (2014–2019)	Total cost (2014–2019)	Total government cost (2014–2019)	Trend
Prednisolone	16,390,059	$253,257,180	$96,718,235	Up
Betamethasone	8,616,640	$266,088,884	$159,901,957	Up
Allopurinol	6,625,962	$89,269,641	$37,871,101	Up
Triamcinolone	4,905,834	$70,161,998	$29,960,557	Up
Methylprednisolone	3,911,183	$92,643,240	$40,556,629	Up
Prednisone	3,687,206	$48,707,232	$19,615,930	Stable
Dexamethasone	3,666,197	$63,549,170	$30,930,017	Stable
Hydrocortisone	2,436,800	$41,382,170	$19,657,190	Down
Amiodarone	1,987,962	$33,713,930	$21,184,186	Up
Fluorometholone	1,408,451	$23,797,616	$8,842,284	Stable
Tamoxifen	877,558	$26,296,362	$10,981,787	Up
Raloxifene	506,690	$24,104,552	$18,996,743	Down
Haloperidol	451,204	$7,660,017	$5,089,181	Stable
Beclomethasone	280,818	$8,992,458	$4,729,624	Down
Cortisone	205,460	$4,539,133	$2,355,101	Stable
Fludrocortisone	162,601	$6,578,401	$4,403,161	Up
Busulfan	1,768	$148,476	$132,684	Stable

**FIGURE 1:**
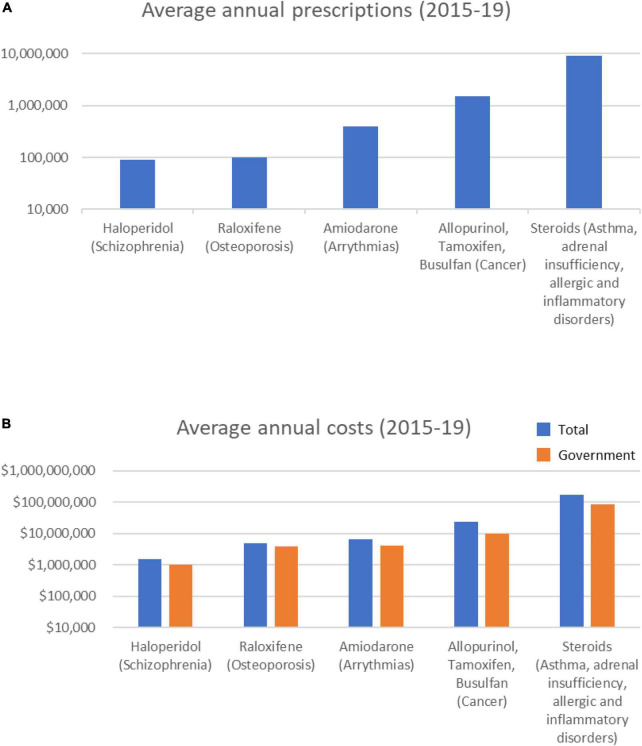
Average annual prescription numbers **(A)** and costs **(B)** for members of the Known category of drugs associated with cataract, together with typical conditions for which they are prescribed.

Most notable among the Known category drugs prescribed over the 5 years were 11 steroids, of which 8 were in the top 10 most prescribed Known drugs (Table 1). On average, each year over 9 M total steroid prescriptions were supplied at a combined average annual cost of > $175 M (Figure 1). Prednisolone (>3 M prescriptions and > $50 M per year) and betamethasone (>1.7 M prescriptions and > $53 M per year) were the most prescribed and also the most costly drugs in the Known category.

Over the 5-year period, 8 Known category drugs showed increasing annual prescriptions (5 steroids) and 9 showed increasing annual costs (Supplementary Figure 1). The total number of annual steroid prescriptions increased 7.7% over the 5 years (9,031,988 in 2014/15 to 9,725,044 in 2018/19), and the associated costs increased 42.9% $142M in 2014/15 to $203M in 2018/19*).*

Overall, these data indicate that numerous Known cataract-inducing drugs are being increasingly used. At a combined average annual cost of >$212 M per year, of which the direct government cost is > $102 M, it is clear a large investment is being made to treat patients using drugs known to increase the rate of cataract formation.

### Probable cataract-inducing drugs largely involves methotrexate

**FIGURE 2:**
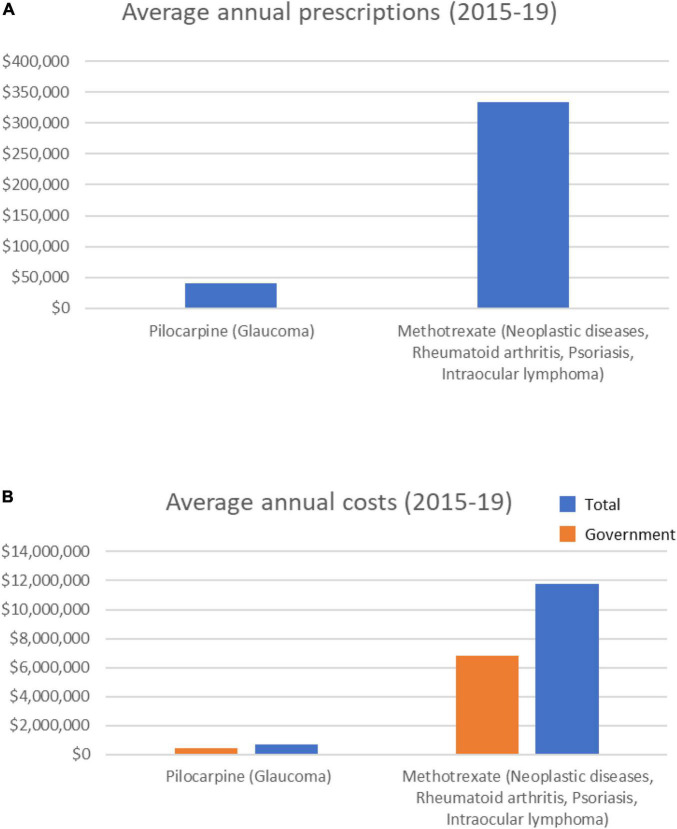
Average annual prescription numbers **(A)** and costs **(B)** for members of the probable category of drugs associated with cataract, together with typical conditions for which they are prescribed.

Of the 9 drugs classified as having a probable association with cataract formation (Supplementary Table 1), only two were prescribed over the 5-year period analyzed–methotrexate and pilocarpine (Table 2). These drugs are prescribed for conditions including cancer and rheumatoid arthritis (methotrexate), or glaucoma (pilocarpine). Methotrexate averaged > 330,000 annual prescriptions (Figure 2), with prescriptions increasing ∼28% over the 5 years (from 298,589 in 2014/15 to 398,799 in 2018/19). Average methotrexate costs were > $11 M/yr (Figure 2), having increased 49% over the 5 years ($9.9M to $14.7M; Supplementary Figure 2). In contrast, pilocarpine, averaged > 40,000 prescriptions/year at an average annual cost of $677,000/year (Figure 2). The prescribing rate for pilocarpine steadily decreased over the 5 years (Supplementary Figure 2).

**TABLE 2:** PBS data (2014–2019) showing prescription numbers and costs for members of the probable category of drugs associated with cataract.

Drug name	Total prescriptions (2014–2019)	Total cost (2014–2019)	Total government cost (2014–2019)	Trend
Methotrexate	1,671,826	$58,958,809	$33,952,887	Up
Pilocarpine	201,535	$3,389,391	$2,168,548	Down

### Possible cataract-inducing drugs dominated by psychotropic drugs

**FIGURE 3:**
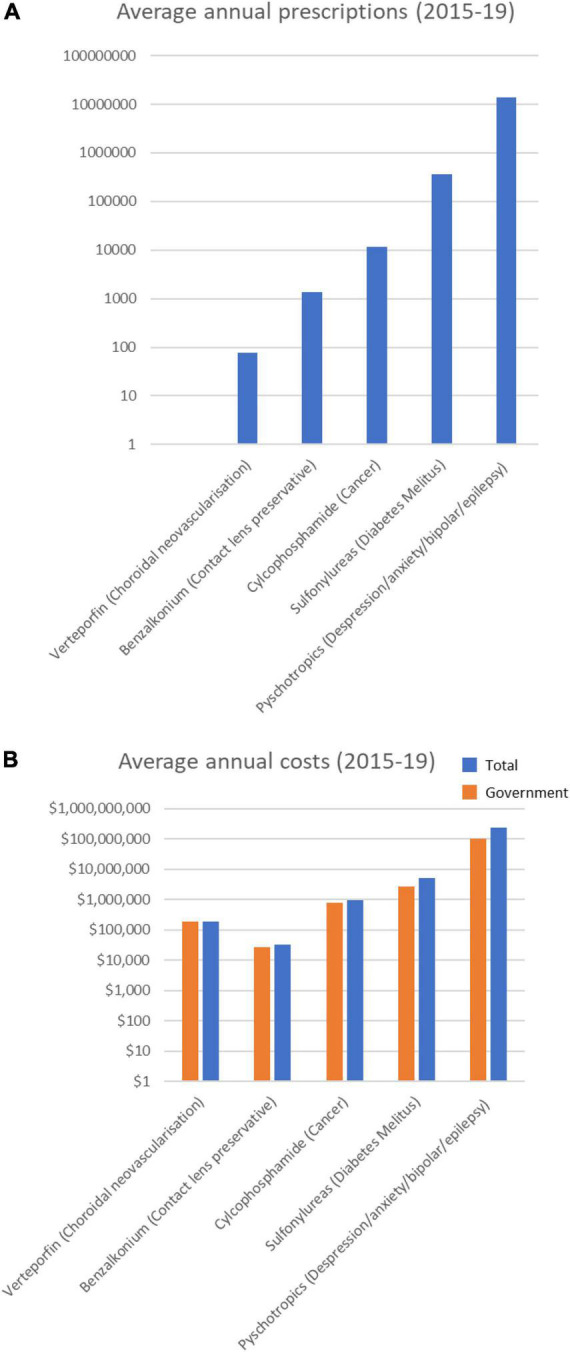
Average annual prescription numbers **(A)** and costs **(B)** for members of the Possible category of drugs associated with cataract, together with typical conditions for which they are prescribed.

Of the 23 drugs having a possible association with increased risk of cataract (Supplementary Table 1), 14 were prescribed over the 5 years (Table 3). These drugs are typically prescribed for conditions from depression to diabetes mellitus. Notably, 8 psychotropic drugs were in the top 10 most prescribed drugs in this category (Table 3). The top 6 most prescribed Possible category drugs averaged more than 1 M prescriptions per year and cost from $16 M to $53 M annually (Figure 3). Psychotropic drug prescriptions averaged > 14 M annually, at a combined average annual cost of >$237 M. Escitalopram (>3.9 M prescriptions and > $53 M per year) and sertraline (>3.9 M prescriptions and >$50 M per year) were the most prescribed and most costly drugs.

**TABLE 3:** PBS data (2014–2019) showing prescription and cost data for drugs that have a probable association with cataract; drugs with gray background are psychotropic drugs (gray background = psychotropic drugs).

Drug name	Total prescriptions (2014–2019)	Total cost (2014–2019)	Total government cost (2014–2019)	Trend
Escitalopram	19,668,355	$267,239,697	$77,242,077	Up
Sertraline	19,621,073	$250,805,008	$75,720,013	Up
Fluoxetine	9,175,210	$159,591,755	$56,952,481	Up
Citalopram	8,757,519	$107,923,398	$36,708,280	Stable
Quetiapine	5,208,959	$250,578,382	$199,228,874	Up
Paroxetine	5,186,599	$84,724,396	$33,597,402	Stable
Fluvoxamine	2,181,096	$47,293,012	$21,200,592	Stable
Glimepiride*	1,111,627	$13,956,211	$6,955,193	Down
Phenytoin	636,940	$21,170,351	$14,014,774	Down
Glibenclamide*	562,997	$9,342,018	$5,090,815	Down
Glipizide*	139,061	$2,416,335	$1,464,987	Down
Cyclophosphamide	57,541	$4,683,890	$3,760,884	Stable
Benzalkonium	6,913	$156,968	$134,716	Down
Verteporfin	391	$940,222	$932,472	Down

*Sulphonylurea drug category.

Of the 14 drugs in the Possible Category, 4 (all psychotropic drugs) increased in annual prescriptions, government cost and total cost, including 4 of the top 5 drugs (Supplementary Figure 3). The other 4 psychotropic drugs showed relatively stable prescribing rates over the past 5 years. Overall, the number of annual psychotropic prescriptions increased by 19.7% over the 5 years (from 12.8 M in 2014/15 to 15.4 M in 2018/19), with an associated increase in costs 7% (from $230 M in 2014/15 to $246 M in 2018/19).

The other drugs having a Possible association with cataracts were the sulfonylureas (Table 3) typically used to treat diabetes mellitus. Sulfonylureas were prescribed at an annual rate of > 360,000 prescriptions a year (Figure 3), costing ∼$5 M/year, and with a steadily decreasing prescribing rate over the 5 years (Supplementary Table 2).

### Statins dominate the drugs with uncertain effects on cataract: Statins

**FIGURE 4:**
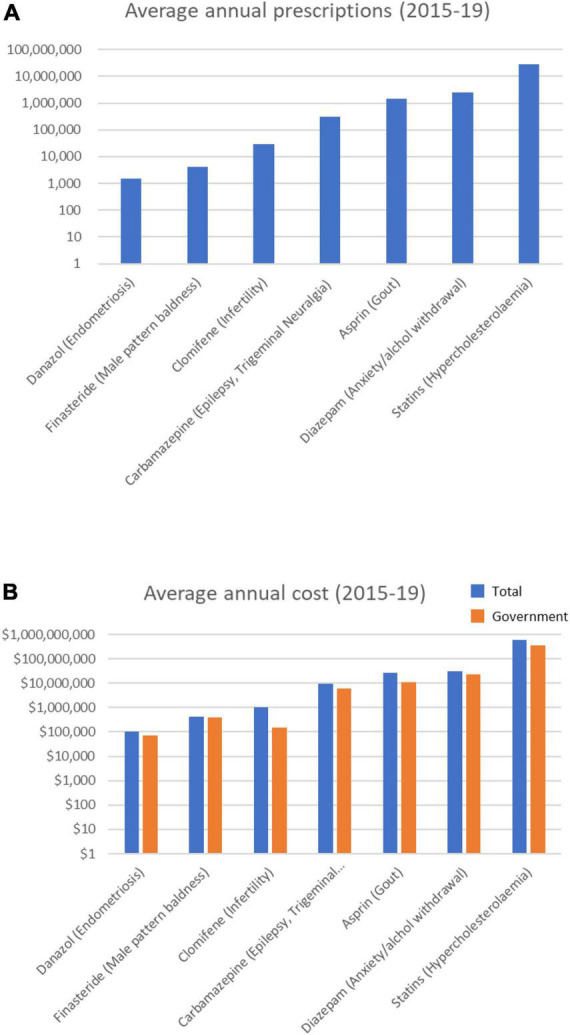
Average annual prescription numbers **(A)** and costs **(B)** for members of the Uncertain category of drugs associated with cataract, together with typical conditions for which they are prescribed.

Of the 15 drugs in the Uncertain category (Supplementary Table 1), the PBS data revealed 11 were prescribed over the 5-year period (Table 4). These drugs are prescribed for conditions ranging from hypercholesterolemia to endometriosis. The top three most prescribed averaged > 3 M annual prescriptions, and cost from $107 M to $260 M a year each (Figure 4). Most notable among these Uncertain category drugs were 5 statins, 3 of which were in the top five most prescribed (Table 4). Atorvastatin (> 12 M prescriptions and > $224 M per year) and rosuvastatin (>10 M prescriptions and > $260 M per year) were the most prescribed and most costly over the 5-year period. On average each year > 27 M statin prescriptions were filled in total at a combined average annual cost of over $604 M.

**TABLE 4:** PBS data (2014–2019) showing prescription numbers and costs for members of the uncertain category of drugs associated with cataract (gray background = statins).

Drug name	Total prescriptions (2014–2019)	Total cost (2014–2019)	Total government cost (2014–2019)	Trend
Atorvastatin	60,343,650	$1,122,411,672	$613,870,024	Stable
Rosuvastatin	54,192,851	$1,301,723,120	$699,934,742	Stable
Simvastatin	19,661,920	$536,558,720	$375,715,069	Down
Diazepam	12,410,290	$136,617,166	$55,009,989	Stable
Aspirin	7,058,298	$159,273,025	$111,798,669	Down
Pravastatin	3,926,680	$56,291,429	$29,753,960	Down
Carbamazepine	1,503,278	$48,796,399	$29,920,620	Stable
Fluvastatin	179,164	$7,182,544	$5,010,457	Down
Clomifene	149,657	$5,227,406	$745,483	Down
Finasteride	21,424	$2,066,376	$1,979,100	Down
Danazol	7,362	$525,664	$355,747	Stable

The prescribing rates of statins were mixed, with some trending up and others trending down over the 5 years (Supplementary Figure 4). Statins were the highest prescribed and most costly drugs in this study (>138 M prescriptions and > $3 billion over 5 years). Overall, the number of annual statin prescriptions increased by 11.8% over the 5 years (from 26,064,117 in 2014/15 to 29,127,562 in 2018/19), though the total costs decreased by –33% (from $775M in 2014/15 to $516M in 2018/19). The annual governments costs decreased by 51% (from ∼$510M in 2014/15 to ∼$249M in 2018/19).

## Discussion

The analysis of PBS data presented here shows tens-of- millions of prescriptions are filled every year in Australia for drugs known to or suspected of inducing cataract. The most prescribed and costly of these drugs are glucocorticoids, psychotropic drugs, and statins.

### Glucocorticoids

Glucocorticoids—a class of drug known to cause posterior subcapsular cataract, particularly in people above the age of 40 (3)—are prescribed for a variety of diseases including ocular conditions (e.g., macula edema), asthma, arthritis and inflammatory bowel disease (4, 5). The data here showed an average of ∼9 M annual steroid prescriptions in Australian over the 5 years from 2014 to 2019. Over that period, the annual number of steroid prescriptions increased 7.7% to 9,725,044 in 2018/19, and the associated costs increased by 42.9% to $203M. A UK trial found, on average, patients require 6.5 glucocorticoid prescriptions (6), and approximately 50% of these patients were 45 years and above (7). Using this as a guide, this equates to ∼1.5 M Australians using prescription steroids in 2018/19 with approximately 750,000 patients 45 years or older. In Australia, the increased risk of PSC due to glucocorticoids is OR 2.5 for inhaled and OR 4.1 for oral corticosteroids. Glucocorticoids were also associated with an increase in nuclear cataracts, with an OR of 2.0 for inhaled and 3.5 for oral corticosteroids (mean age 63 years) (3).

As discussed below, minimizing drug-induced cataract could help offset the burden of cataract in Australia and elsewhere. The large number and cost of cataract surgeries likely arising from prescription steroids provides a strong argument for better understanding the molecular mechanisms of steroid-induced cataract. Application of such molecular knowledge could lead to early identification of at-risk patients, and potentially to development of alternative treatment strategies. For example, folate is recommended as a co-therapy with methotrexate prescribed for rheumatoid arthritis, to mitigate risks associated with methotrexate-induced folate deficiency (8).

Clinically, the cataracts caused by glucocorticoid steroids are centrally-located, posterior subscapular cataracts with vacuoles (9, 10)—suggesting they relate to aberrant migration of lens epithelial cells (LECs) along the posterior capsule. At present, there is little data describing the molecular mechanisms of steroid-induced cataracts in human lenses. While steroids can bind to lens proteins, this is generally discounted as a mechanism for cataract formation as they do so with lower affinity than other proteins that do not induce cataract (11).

Primary human LECs transfected with firefly luciferase (controlled by glucocorticoid response elements) showed increased luciferase activity when exposed to dexamethasone (12)—demonstrating the ability of primary human LECs to activate the glucocorticoid receptor. In the same study, microarray analysis of the immortalized human LEC line, HLE B-3, after dexamethasone treatment revealed altered expression of various genes (136 and 86 genes after 4 and 16 h of treatment, respectively).

Analysis of primary human LECs from patients with steroid-induced cataract showed a small increase in mRNA expression and protein activity for the matrix metalloproteinases, MMP2 and MMP-9 (13). Additional studies using primary and immortalized human LECs showed dexamethasone treatment led to phosphorylation of the glucocorticoid receptor, altered expression of MAPK and PI3K/AKT regulators, decreased phosphorylation of MAPK- and AKT-related proteins (14), and altered expression of cell adhesion molecules (15). No detectable effect of dexamethasone on proliferation or apoptosis of the human LEC line (HLE B-3) was observed, though some dexamethasone-induced apoptosis in human lens cells has been reported elsewhere (16, 17).

Overall, these studies provide initial insights into steroid-induced effects in human lens cells. However, their clinical relevance remains unclear due to differences (abnormalities) in behavior of immortalized lens cells compared to normal primary human LECs, and also the short timeframes being analyzed (i.e., typically < 24 h). Notably, none of these *in vitro* studies involving human LECs or lens cell lines were able to assess the effects of dexamethasone on critical lens functional properties of transparency or focusing.

### Psychotropic drugs

In Australia, one in five people experienced a mental disorder in a 12-month period (18), with many of them prescribed psychotropic drugs. The PBS data show eight psychotropic drugs from the possible category were prescribed in Australia, with most averaging > 1 M prescriptions a year. The average annual number of psychotropic drug prescriptions was > 14 M, with an associated cost of > $237 M per year. Annual prescriptions increased 19.7% over the 5 years to 15.4 M in 2018/19, at a cost of $246 M (a 7% increase over the 5 years).

Published health record analyses have shown significant positive associations between risk of cataract formation and use of psychotropic drugs, including: citalopram (OR = 1.53, 95% CI, 1.33–1.77; *P* < 0.001) and fluvoxamine (RR, 1.39; 95% CI, 1.07–1.80) (19, 20); fluoxetine (AOR: 1.21; 95% CI, 1.01–1.46, *p* = 0.042) (21), fluvoxamine (AOR: 1.47; 95% CI: 1.01–2.12, *p* = 0.043) and sertraline (AOR: 1.29; 95% CI, 1.12–1.48, *p* < 0.001); sertraline (AOR: 1.29; 95% CI, 1.12–1.48, p < 0.001) and fluvoxamine (AOR: 1.37; 95% CI, 1.07–1.76, *p* = 0.012) (21). A recent meta-analysis also identified an association between cataract and use of fluoxetine (RR 1.08, 95% CI, 1.03–1.12) and fluvoxamine (RR 1.22, 95% CI, 1.06–1.40) (22).

These studies also showed many working-age patients (e.g., 50–64) who have taken these psychotropic drugs have required cataract surgery, with direct implications for labor force productivity (discussed below) as well as direct medical costs. In some studies, the average time to cataract diagnosis while on SSRI (Selective Serotonin Reuptake Inhibitor) therapy was relatively short, ∼656 days (20). The large annual number of psychotropic prescriptions in Australia may lead to sizeable numbers of drug-induced cataract.

At present, it is not clear how psychotropic drugs could lead to cataract formation in patients. It is possible selective serotonin reuptake inhibitors (such as citalopram, escitalopram, fluoxetine, fluvoxamine and sertraline all prescribed in Australia) could lead to elevated levels of serotonin, as detected in the aqueous humor of patients having undergone cataract surgery (23). In rats, application of serotonin *via* injection or eyedrops led to rapid development of dense cataracts thought to be related to reduced aqueous (24). These findings suggest an indirect mechanism for cataract formation *via* selective serotonin reuptake inhibitors. However, rabbit lenses express serotonin receptors (e.g., 5-HT_1A_ and 5-HT_7_) (25) and exposure of rabbit LECs to serotonin led to phosphoinositide turnover (26). A more comprehensive analysis of the effects of psychotropic drugs on human cataract formation is needed.

### Statins

Statins are prescribed to reduce blood cholesterol levels in order to reduce the risk of cardiovascular disease. Statins are effective inhibitors of 3-hydroxy-3-methyl-glutaryl-CoA reductase, a key enzyme in cholesterol biosynthesis. Statins can also increase expression of low density lipoprotein (LDL) receptors, enabling liver cells to capture cholesterol-containing LDL particles from the blood (27). The PBS data revealed the average annual number of statin prescriptions in Australia is > 27 M, with an average annual cost of > $604 M. Approximately 44% of Australians were prescribed and used statins in 2016 (28). Statins are listed in the Uncertain category of cataract-inducing drugs. In the lens, it appears cholesterol levels need to be maintained within a relatively narrow range to avoid cataract formation (29). Increased cholesterol levels in the lens–for example, 25-hydroxycholesterol–have been associated with cataract (30). Conversely, the cholesterol-lowering drug, triparanol, also causes irreversible cataract (31). Triparanol inhibits cholesterol synthesis downstream of lanosterol production, leading to accumulation of lanosterol in lenses (32, 33). While recent reports suggest cataracts can be dissolved with intermediates of cholesterol biosynthesis–lanosterol or 25-hydroxycholesterol (34, 35) (that increase the chaperone activity of α-crystallin)–other studies have failed to replicate these effects anti-cataract effects (30, 36, 37). Perhaps unsurprisingly, the evidence relating to statins and cataract formation has been contradictory. Outcomes from systematic reviews have been mixed–with positive, negative and no associations all identified (38–41). A recent meta-analysis encompassing 313,200 patients found no association with cataract. However, given the heterogeneity in the studies underpinning the meta-analysis, it was recommended that large, multicenter, pragmatic, prospective observational studies or registries be performed to assess the risk of cataracts arising from statin use (41).

### Elucidating mechanisms of drug-induced cataracts in humans

Worldwide, cataracts are a large and increasing cause of blindness. The number of people with low vision or blindness due to cataracts increased from 50.5 M in 1990 to 65.2 M in 2015 (1), because of the increasing size and age of populations worldwide. The PBS data analysis presented here indicates prescription drugs could be a significant source of cataracts in Australia. In the United States, outpatient services are used annually by 1.6 M cataract patients aged 40-64 years, and 8.9 M aged 65 and older (42). Direct medical costs attributed to these two groups are $2.14 billion/year and $4.66 billion/year, respectively. US cataract patients also contributed to $11.2 billion in other annual direct costs (e.g., care programs); and $8 billion in annual productivity losses (e.g., lower participation and lower wages). Worldwide, the economic, employment and social consequences of cataracts cost $tens-of-billions annually. It is possible a significant proportion of annual cataract cases arise due to prescription drug use in both working-aged people and retirees. However, cataractous human lens tissue is difficult to obtain, and transparent/light-focusing human lens tissue is essentially impossible to reliably obtain in meaningful amounts for research during the early stages of cataract formation.

Human pluripotent stem cells offer the ability to generate large numbers of human LECs and light-focusing micro-lenses (43, 44). These stem cell-derived human LECs share morphological, transcriptional and proteomic profiles similar to fetal human LECs (43, 44). Light-focusing micro-lenses derived from these human LECs share similar anatomical and molecular characteristics with human lenses, including expression of a broad range of crystallin proteins associated with the focusing ability of primary human lenses (43, 44). Notably, exposing human stem cell-derived micro-lenses to a cystic fibrosis drug suspected of causing cataract in human patients (43, 44), or to dexamethasone (45), resulted in decreased light focusing in the treated micro-lenses. Together, these data suggest human stem cell-derived micro-lenses may provide a useful new tool for investigating the initiating molecular mechanisms of drug-induced cataract. Consistent with this, the human micro-lens system is amenable to detailed analyses including imaging (light, confocal and electron microscopy), transcriptomics and proteomics. Thus, the human micro-lens system provides a novel and potentially powerful approach to time-course cataract studies *in vitro*, with lens transparency and light-focusing as functional end-points. This includes new opportunities to elucidate the molecular mechanisms through which prescription drugs cause cataracts. Such new knowledge could provide opportunities to decrease the annual global burden of cataract through improved identification of at-risk patients, prescription of co-therapies, or identification of candidate anti-cataract drugs. Such studies would address the large, unmet need for a reduction in the amount of drug-induced cataract that currently occurs worldwide. Use of human stem cell-derived micro-lenses could also provide a functional human lens system to reduce reliance on animal-based lens models for investigating molecular mechanisms of cataract formation (46, 47).

## Materials and methods

Public PBS drug prescription data from Australia was analyzed for the period 2014 to 2019, to identify the prescribing rates for drugs associated with cataract formation. Categories of drugs that have different associations with cataract formation were obtained from Drug-Induced Ocular Side Effects, 7th Edition. Australian PBS data supply records were downloaded from PBS and RPBS Section 85 Date of Supply Data (48). This data was divided into financial years from July to June based on the month of supply on the PBS per item. Item codes for drugs of interest were matched with the item codes in the PBS prescription record data. Total frequency of prescriptions, cost and government cost for the July–June financial years from 2014 to 2019. The frequency of total prescriptions, average annual prescription rates, total cost, average annual cost and total government cost were calculated for each potential cataract causing agent.

## Data availability statement

Publicly available datasets were analyzed in this study. This data can be found here: Health AGD of Pharmaceutical Benefits Scheme (PBS) and PBS and RPBS Section 85 Date of Supply Data.

## Author contributions

MO’C: conceptualization, resources, project administration, and funding acquisition. MO’C and KM: methodology and supervision. JC and MO’C: validation and visualization. JC: formal analysis, investigation, and writing—original draft preparation. JC, MO’C, and KM: writing—review and editing. All authors have read and agreed to the published version of the manuscript.

## Abbreviations

AOR: adjusted odds ratio
CI: confidence interval
LEC: lens epithelial cell
M: million
PBS: Pharmaceutical Benefits Scheme
RR: relative risk.
