# Supplementary material for: Drugs associated with cataract formation represent an unmet need in cataract research
Source: Front Med (Lausanne). 2022 Aug 15;9:947659. doi: 10.3389/fmed.2022.947659 (PMC9420850; doi:10.3389/fmed.2022.947659)
Supplement: Supplementary file 2 [file Table_2.docx]

Supplementary table 2: Trend of sulfonylurea prescriptions over 2014-2019.
