# Supplementary figures and images for: Drugs associated with cataract formation represent an unmet need in cataract research
Source: Front Med (Lausanne). 2022 Aug 15;9:947659. doi: 10.3389/fmed.2022.947659 (PMC9420850; doi:10.3389/fmed.2022.947659)

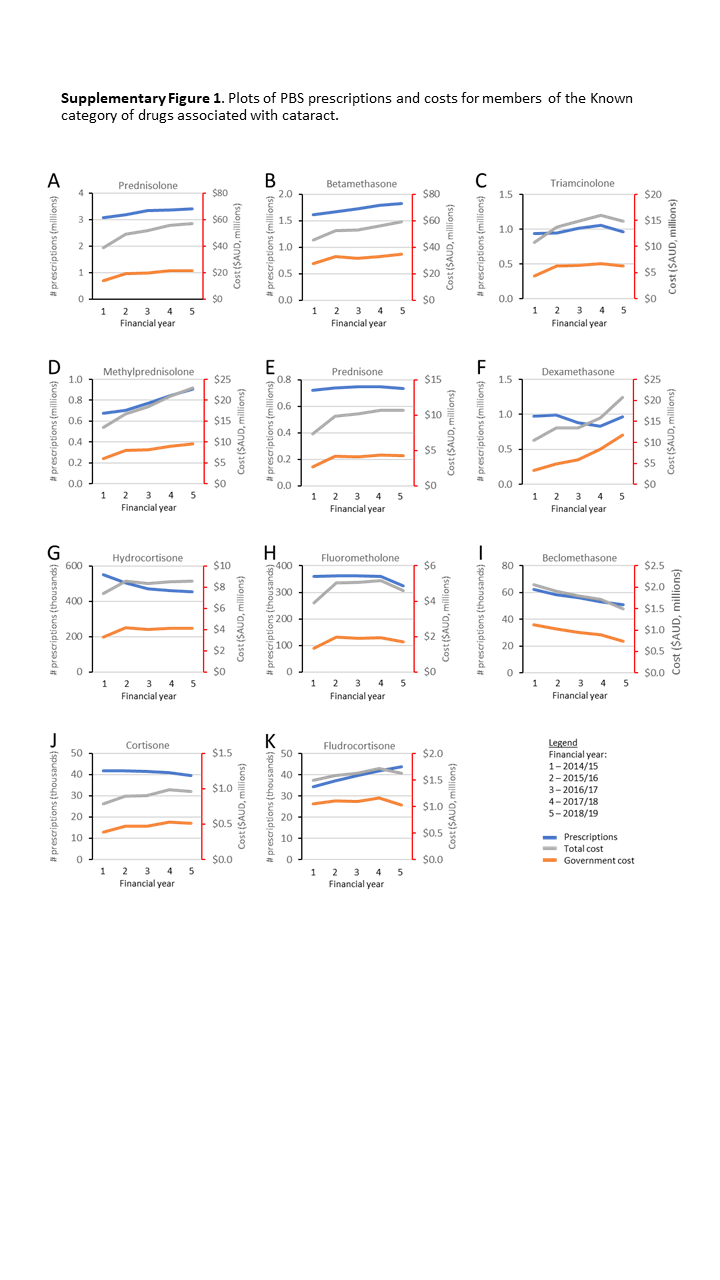

Supplement: Supplementary file 3 [file Image_1.TIF]

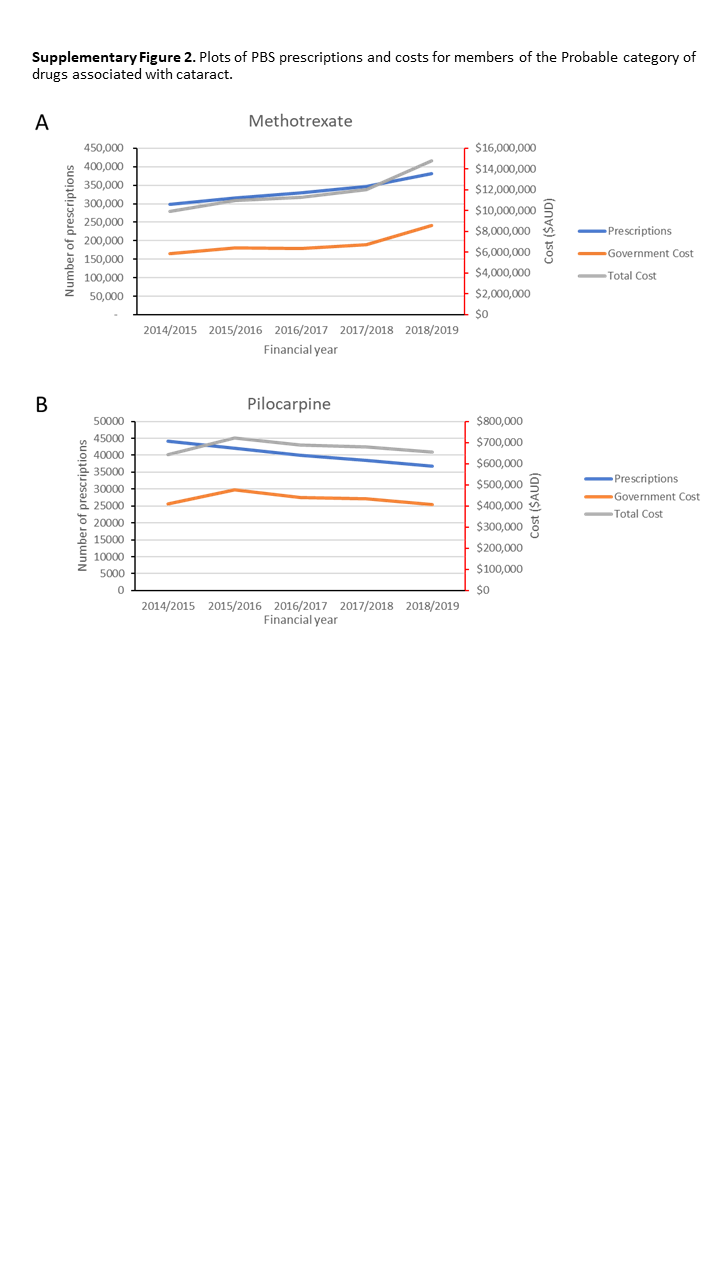

Supplement: Supplementary file 4 [file Image_2.TIF]

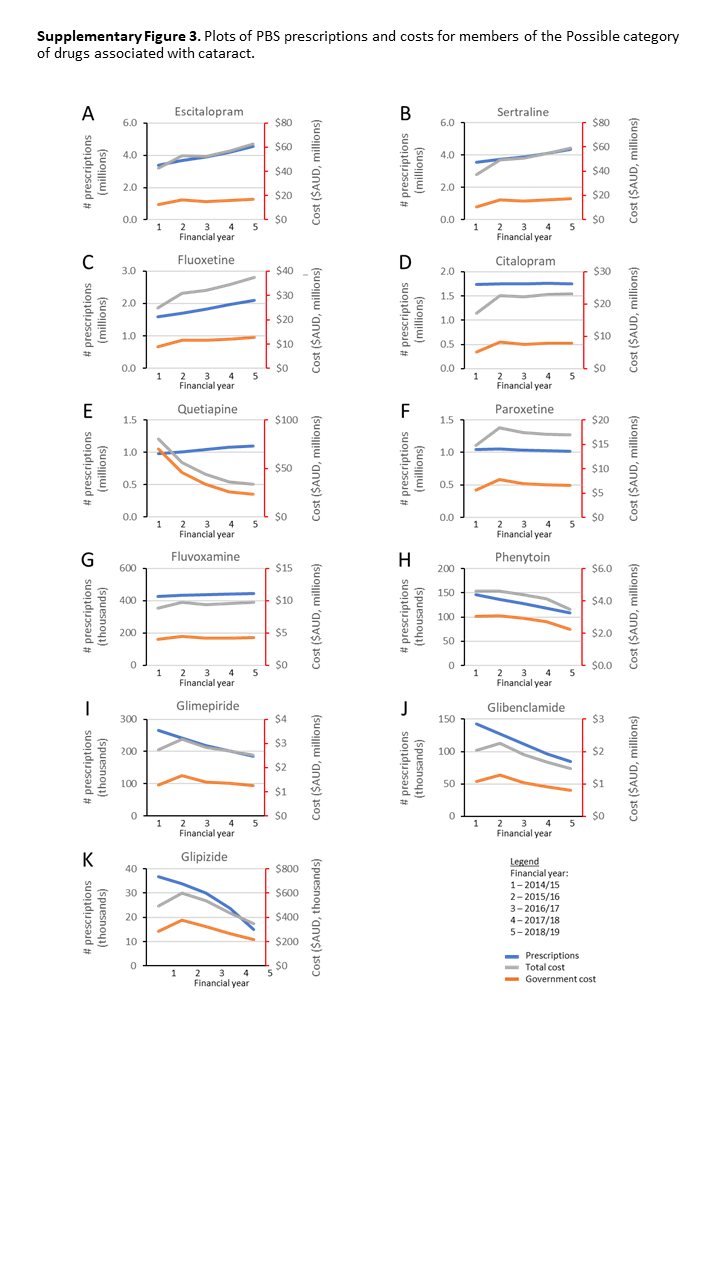

Supplement: Supplementary file 5 [file Image_3.TIF]

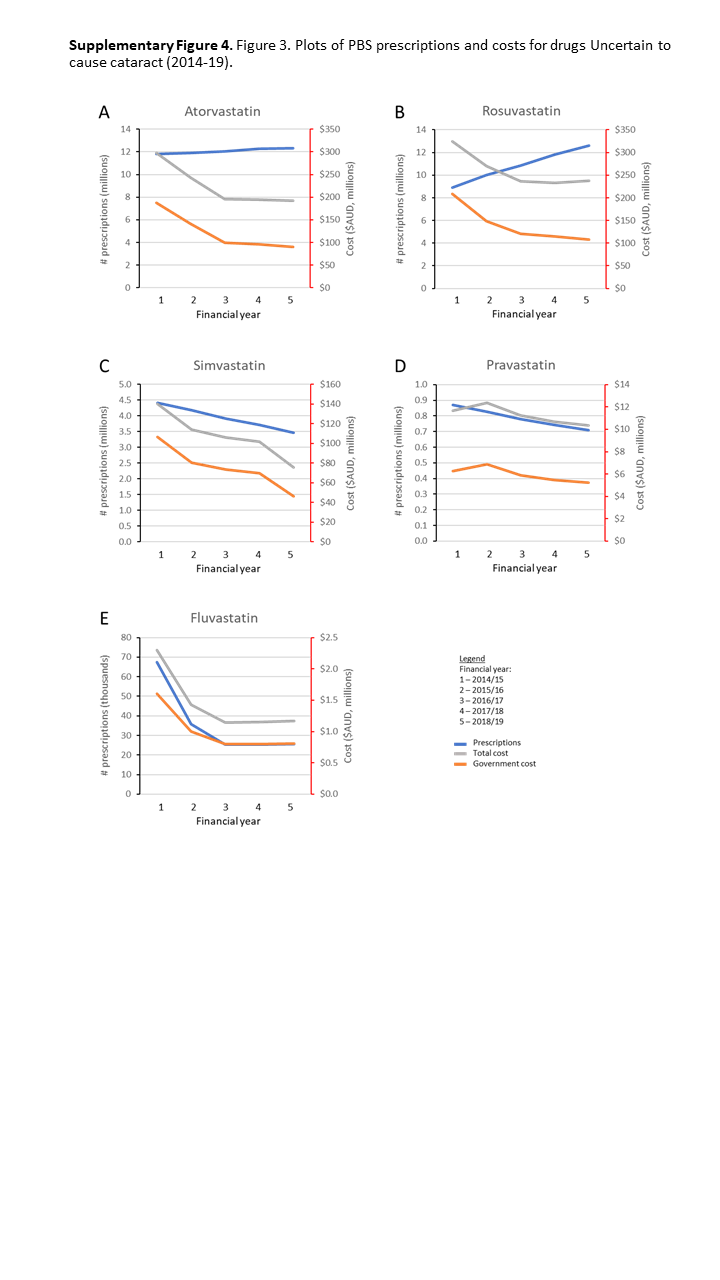

Supplement: Supplementary file 6 [file Image_4.TIF]
