# Supplementary material for: Drugs associated with cataract formation represent an unmet need in cataract research
Source: Front Med (Lausanne). 2022 Aug 15;9:947659. doi: 10.3389/fmed.2022.947659 (PMC9420850; doi:10.3389/fmed.2022.947659)
Supplement: Supplementary file 3 [file Table_1.docx]

| Known | Probable | Possible | Uncertain |
|---|---|---|---|
| Prednisolone | Methotrexate | Escitalopram | Atorvastatin |
| Betamethasone | Pilocarpine | Sertraline | Rosuvastatin |
| Allopurinol | | Fluoxetine | Simvastatin |
| Triamcinolone | | Citalopram | Diazepam |
| Methylprednisolone | | Quetiapine | Aspirin |
| Prednisone | | Paroxetine | Pravastatin |
| Dexamethasone | | Fluvoxamine | Carbamazepine |
| Hydrocortisone | | Glimepiride | Fluvastatin |
| Amiodarone | | Phenytoin | Clomifene |
| Fluorometholone | | Glibenclamide | Finasteride |
| Tamoxifen | | Glipizide | Danazol |
| Raloxifene | | Cyclophosphamide | |
| Haloperidol | | Benzalkonium | |
| Beclomethasone | | Verteporfin | |
| Cortisone | | | |
| Fludrocortisone | | | |
| Busulfan | | | |
